# Adherence to pancreatic enzyme replacement therapy among patients with chronic pancreatitis in East China: a mixed methods study

**DOI:** 10.1038/s41598-023-44519-3

**Published:** 2023-10-10

**Authors:** You Zhou, Ren-Qian Huang, Qi-Wei Wu, Jin-Jie Xu, Jin-Hui Yi, Cui Chen, Guo-Tao Lu, Zhao-Shen Li, Dan Wang, Liang-Hao Hu

**Affiliations:** 1 Department of Gastroenterology, Changhai Hospital, Naval Medical University, Shanghai, China; 2 School of Nursing, Chinese Academy of Medical Sciences & Peking Union Medical College, Beijing, China; 3 Faculty of Nursing, School of Medicine, Zhejiang University, Hangzhou, China; 4 Pancreatic Center, Department of Gastroenterology, Affiliated Hospital of Yangzhou University, Yangzhou University, Yangzhou, China

**Keywords:** Chronic pancreatitis, Patient education, Risk factors

## Abstract

Pancreatic enzyme replacement therapy (PERT) has been recommended as the preferred method for pancreatic exocrine insufficiency caused by chronic pancreatitis (CP). However, at present, the patient-related factors for the poor PERT management are not clear, and there are no studies on the adherence to PERT in patients with CP in East China. This was a mixed-method study following the principle of sequential explanatory design and included two parts: a quantitative and qualitative study. A cross-sectional survey of medication adherence (MA) was first carried out, followed by a semi-structured interview to further explore and explain the influencing factors of adherence to PERT. Of the 148 patients included in this study, 48.0% had poor MA and only 12.8% had good MA. Multivariate logistic regression showed that lower levels of education and income were contributing factors for non-adherence to PERT. Semi-structured interviews with 24 patients revealed that the reasons for non-adherence also included lack of knowledge, self-adjustment of PERT, lifetime of medication, side effects of PERT, forgetfulness, financial burdens, and accessibility issues. The adherence to PERT was poor among patients with CP in East China. Healthcare providers should personalize medication strategies to improve patients’ MA.

## Introduction

Chronic pancreatitis (CP) is a rare progressive inflammatory disease of the pancreas characterized by irreversible pancreatic acinar atrophy and fibrosis^1^. The global incidence of CP is about 4.4–14.0 per 100,000 people, the prevalence is about 36.9–52.4 per 100,000 people, and there is a trend of increasing year by year^2,3^. The main symptoms of CP patients are chronic abdominal pain and pancreatic endocrine and exocrine loss symptoms such as elevated blood sugar, maldigestion, and weight loss. In addition, patients may also have complications such as pancreatic duct stones, pancreatic duct stenosis, and pancreatic cysts and have a higher risk of pancreatic cancer, which significantly affects their quality of life^4,5^.

Pancreatic exocrine insufficiency (PEI) is one of the common complications of CP, and its incidence increases progressively with the development of disease and prolongation of course^6^. Steatorrhea occurs when pancreatic enzyme production decreases by more than 90%^7^. PEI is associated with maldigestion and significantly affects the absorption of protein, fat, micronutrients, and fat-soluble vitamins, resulting in nutritional deficiencies^8,9^, complications such as osteoporosis^10^, adverse cardiovascular events^11^, infections^12^ and an increased risk of death^13^. Guidelines have recommended pancreatic enzyme replacement therapy (PERT) as the cornerstone and preferred treatment method for PEI to improve malabsorption and malnutrition and prevent their adverse outcomes in CP patients^14,15^. However, studies have shown that the current status of treatment for PEI in CP patients is not ideal: there are significant insufficiencies in the diagnosis and treatment for PEI and there is still much room for improvement in PERT management^6,16–18^.

Medication adherence (MA) refers to “the extent to which patients take medications as prescribed by their health care providers”^19^. Great MA plays an important role in disease control, symptom relief, and improving prognosis^20^. The status of patients’ MA has been extensively explored in a wide range of other chronic diseases. However, at present, research on patients’ adherence on PERT in pancreatic diseases is still quite limited. Of the available research evidence, Barkin et al. found that the adherence rate of PERT in pancreatic cancer patients was 50.1%^21^. A US study of patients undergoing total pancreatectomy with islet autotransplantation found a 79% adherence rate of PERT after surgery^22^. A retrospective study by Khandelwal et al. discovered that the PERT adherence rate in patients with PEI caused by CP, pancreatic cancer and pancreatectomy was 48%, 52% and 52%, respectively, and this figure declined rapidly to about 20% within a one-year follow-up period^23^. These data were all from Western countries, to date, there are no studies on adherence to PERT in Chinese CP patients. The influencing factors of adherence to PERT among patients with CP in China are still unclear.

It is of great significance to identify the status and factors of adherence disorder of PERT for the individualized formulation of medication strategies and to improve the level of patients’ MA. Therefore, the aims of this study were to (1) clarify the status of adherence to PERT among patients with CP in East China, and (2) explore the influencing factors of adherence to PERT to provide reference and inspiration for PERT management among CP patients.

## Materials and methods

### Study design

This was a cross-sectional study based on a mixed-method approach conducted at First Affiliated Hospital of Naval Medical University (Changhai Hospital) in Shanghai, China, including two parts: a quantitative and qualitative study. Following the principle of sequential explanatory design, quantitative analysis was conducted first, followed by in-depth interviews to explore and explain various subjective and objective influencing factors to adherence to PERT among Chinese CP patients from a more comprehensive perspective.

This study protocol was approved by the Changhai Hospital Ethics Committee (CHEC2020-026) and registered on ClinicalTrial.gov (NCT05489003). The study was conducted following the Declaration of Helsinki, and all patients signed informed consent.

### Quantitative study

#### Data source, sampling method, and eligibility criteria

As part of an ongoing prospective study of symptoms and quality of life among patients with CP in China, this study included all CP patients admitted to the Department of Gastroenterology in our hospital from June to September 2022. The diagnosis of CP conformed to the Asia–Pacific consensus^24^. We excluded patients who (1) were under 18 or over 70 years old, (2) received PERT for less than 3 months, (3) were pregnant, (4) had a comorbid malignancy, (5) had communication disorders, (6) had a history of mental illness or were taking psychotropic drugs, and (7) refused to participate in this study or participated in other relevant studies at the same time.

#### Measurements and data collection

Patients’ MA was measured by the Chinese version of the Morisky Medication Adherence Scale (C-MMAS-8)^25^.

According to literature search and expert opinions, patients’ data were collected through self-designed sociodemographic and clinical characteristics questionnaire. The questionnaire included patient’s age, gender (male or female), education level (junior high school or below, senior high school, university or above), marital status (married, unmarried, divorced, widowed), employment status (employed, unemployed, retired, in study), monthly household income (CNY) (< 5000, 5000–10,000, ≥ 10,000)^26^, residence (urban or rural), type of medical insurance (rural cooperative medical insurance, urban residents’ medical insurance, employee medical insurance), course of disease, type of pain after taking medication (repeat attacks of acute pancreatitis, repeat pain, repeat acute attacks and pain, chronic pain), and history of diabetes, smoking, and drinking (yes or no)^27^.

The MMAS-8 was developed by Morisky et al. in 2008 to evaluate MA of outpatients^28^. The scale consists of 8 items, items 1–4 and 6–7 are scored 0 for “yes” and 1 for “no”, item 5 is reverse scored, and item 8 is scored on a 5-point Likert scale (1: “never”, 0.75: “occasionally”, 0.5: “sometimes”, 0.25: “usually”, 0: “always”). The total MMAS-8 score is the sum of the scores of each item. A score of less than 6 indicates poor MA, greater than 6 and less than 8 indicates moderate MA, and a score of 8 indicates good MA. There were no significant differences between MMAS-8 and C-MMAS-8 in terms of items, contents, and scoring. The validity and reliability of C-MMAS-8 has been validated in a Chinese population^25^.

#### Data analysis

According to the C-MMAS-8 scoring standard, patients were divided into two groups: medication adherence and medication nonadherence, with a cut-off score of 6. Statistical analysis was performed using SPSS 26.0 software. All data were analyzed descriptively. The Kolmogorov–Smirnov test was used to test the normality of data. Continuous variables with normal distribution and skewed distribution and categorical variables were described by mean and standard deviations, median with interquartile range, and frequencies and proportions, respectively. Pearson *χ*^*2*^ test was used for between-group comparisons of categorical variables. Based on the results of univariate analysis, variables with statistically significant differences were selected for multivariate logistic regression analysis to further explore the influencing factors of adherence to PERT. All statistics were two-sided, and *P* values less than 0.05 were considered statistically significant.

### Qualitative study

The qualitative study was designed and conducted following the Consolidated Criteria for Reporting Qualitative Research (COREQ) checklist.

#### Sampling method

To further explore the factors of barriers to adherence to PERT, the interviewed patients were recruited by purposive sampling based on different C-MMAS-8 scores.

#### Data collection

Qualitative data were collected using a semi-structured interview, which was conducted after written consents (including permission to record) were obtained from the patients. The interviews were conducted by two experts with a doctor’s degree and a master’s degree in medicine and nursing, who were experienced in PERT management and qualitative research, respectively, in the form of face-to-face in the meeting room of the ward. Each interview lasted for 25 to 40 min and revolved around five open-ended questions that asked to the participants: “How do you feel about your current MA?”, “How do you take your pancreatic enzyme?”, “How do you feel after taking pancreatic enzyme?”, “Have you encountered any problems and difficulties in the process of taking pancreatic enzyme?”, “What do you think of the current prescription of pancreatic enzyme?”. Field notes were taken throughout the interviews. Data saturation was considered only after no new contents appeared in three consecutive interviews^29^. After 20 interviews, the authors found that no new contents appeared. To check the data saturation, four more interviews were conducted. Finally, the data collection stopped after 24 interviews due to there were no new information and themes obtained in the four additional interviews. To protect patient privacy, during the interviews, only the interviewee and participants were present. All participants were given numerical numbers. Individual information (name, occupation, etc.) that can identify the patient shall be kept by another author.

#### Data analysis

The thematic analysis by Braun and Clarke was used for qualitative data analysis^30^. All records were transcribed and validated by two researchers within 24 h after each interview. Two researchers independently coded the first four transcripts by reading and comparing the notes. After another four coding of transcripts, the initial codebook agreed by the researchers was established. Subsequently, the first researcher continued to code the remaining transcripts and discussed with the second researcher to reach a consensus when new codes appeared. After the coding was completed, the research team organized the coded data into themes. All research results were based on discussions and consensus reached by the entire research team.

### Ethics approval and consent to participate

The study was conducted according to the guidelines of the Declaration of Helsinki and approved by the Changhai Hospital Ethics Committee (CHEC2020-026). All the participants have signed an informed consent.

## Results

### Quantitative analysis

#### Characteristics of participants

A total of 148 patients were recruited for this study, 11 were directly considered to have poor MA because they proactively reported had not continued their medication after their last PERT medication had been exhausted several months earlier, and 137 completed the C-MMAS-8. Table 1 showed the characteristics of the participants. The mean age of the patients was 40.18 ± 13.44 years, and 67.6% (100/148) were male. The mean C-MMAS-8 score was 5.92 ± 1.65 points, 48.0% (71/148) of patients had poor MA, 39.2% (58/148) had moderate MA, and 12.8% (19/148) had good MA.

**Table 1:** Characteristics of participants (n = 148).

Characteristics	n (%)
Age, n (%)	
Mean (SD)	40.18 (13.44)
56–70	25 (16.9)
36–55	68 (46.0)
18–35	55 (37.2)
Gender, n (%)	
Male	100 (67.6)
Female	48 (32.4)
Education level, n (%)	
Junior high school or below	33 (22.3)
Senior high school	42 (28.4)
University or above	73 (49.3)
Marital status, n (%)	
Married	108 (73.0)
Unmarried/divorced/widowed	40 (27.0)
Employment, n (%)	
Employed	88 (59.5)
Unemployed/retired/ in study	60 (40.5)
Monthly household income (CNY), n (%)	
≥ 10,000	33 (22.3)
5000–10,000	53 (35.8)
< 5,000	62 (41.9)
Residence, n (%)	
Urban	101 (68.2)
Rural	47 (31.78)
Medical insurance type, n (%)	
Employee medical insurance	64 (43.2)
Urban residents' medical insurance	41 (27.7)
Rural cooperative medical insurance	43 (29.1)
Course of disease, n (%)	
< 5	76 (51.4)
≥ 5	72 (48.7)
Type of pain after taking medication	
RAP/RAP + RP	83 (56.1)
RP	42 (28.4)
Chronic pain	4 (2.7)
No pain	19 (12.8)
Diabetes, n (%)	50 (33.8)
Steatorrhea, n (%)	46 (31.1)
History of smoking, n (%)	73 (49.3)
History of drinking, n (%)	76 (51.4)
C-MMAS-8 score^a^, n (%)	
Mean (SD)	5.92 (1.65)
< 6	71 (48.0)
6–< 8	58 (39.2)
8	19 (12.8)

CNY, Chinese Yuan; CP, chronic pancreatitis; RAP, repeat attacks of acute pancreatitis; RAP + RP, repeat acute attacks and pain; RP, repeat pain; SD, standard deviations.

*n = 137.

#### Factors associated with medication adherence.

Table 2 showed the results of the univariate analysis. There were significant differences between the two groups in terms of gender (*P* = 0.036), age (*P* = 0.043), education level (*P* = 0.005), employment status (*P* = 0.016), monthly household income (*P* = 0.003), type of health insurance (*P* = 0.014), and history of smoking (*P* = 0.021) and drinking (*P* = 0.033). There was no statistically significant difference in MA between patients with different marital status, residence, course of disease, type of pain after taking medication, diabetes, and steatorrhea.

**Table 2:** Univariate analysis of factors influencing adherence to PERT among patients with CP.

Characteristics	Medication Adherence	Medication Nonadherence	*P* value
	(n = 77)	(n = 71)	
Age, n (%)			
56–70	18 (23.38)	7 (9.86)	**0.043**
36–55	36 (46.75)	32 (45.07)	
18–35	23 (29.87)	32 (45.07)	
Gender, n (%)			
Male	58 (75.32)	42 (59.15)	**0.036**
Female	19 (24.68)	29 (40.85)	
Education level, n (%)			
Junior high school or below	9 (11.69)	24 (33.80)	**0.005**
Senior high school	25 (32.47)	17 (23.94)	
University or above	43 (55.84)	30 (42.25)	
Marital status, n (%)			
Married	60 (77.92)	48 (67.61)	0.158
Unmarried/divorced/widowed	17 (22.08)	23 (32.39)	
Employment, n (%)			
Employed	53 (68.83)	35 (49.30)	**0.016**
Unemployed/retired/in study	24 (31.17)	36 (50.70)	
Monthly household income (CNY), n (%)			
≥ 10,000	25 (32.47)	8 (11.27)	**0.003**
5000–10,000	28 (36.36)	25 (35.21)	
< 5000	24 (31.17)	38 (53.52)	
Residence, n (%)			
Urban	56 (72.83)	45 (63.38)	0.222
Rural	21 (27.27)	26 (36.62)	
Medical insurance type, n (%)			
Rural cooperative medical insurance	16 (20.78)	25 (35.21)	**0.014**
Urban residents' medical insurance	19 (24.68)	24 (33.80)	
Employee medical insurance	42 (54.55)	22 (30.99)	
Course of disease (y), n (%)			
< 5	44 (57.14)	32 (45.07)	0.142
≥ 5	33 (42.86)	39 (54.93)	
Type of pain after taking medication, n (%)			
RAP/RAP + RP	36 (46.75)	47 (66.20)	0.106
RP	27 (35.06)	15 (21.13)	
Chronic pain	3 (3.90)	1 (1.30)	
No pain	11 (14.29)	8 (10.39)	
Diabetes, n (%)	25 (32.47)	25 (35.21)	0.724
Steatorrhea, n (%)	21 (27.27)	25 (35.21)	0.297
History of smoking, n (%)	45 (58.44)	28 (39.44)	**0.021**
History of drinking, n (%)	46 (59.74)	30 (42.25)	**0.033**

CNY, Chinese Yuan; CP, chronic pancreatitis; PERT, pancreatic enzyme replacement therapy; RAP, repeat attacks of acute pancreatitis; RAP + RP, repeat acute attacks and pain; RP, repeat pain; SD, standard deviations.

Significant values are in bold.

Table 3 presented the results of the multivariable logistic regression analysis based on the eight variables that were statistically significant in univariate analysis. Patients with senior high school (adj.OR = 0.18, 95% CI 0.06–0.56, P = 0.003) and university and higher (adj.OR = 0.32, 95% CI 0.11–0.94, P = 0.039) education had lower odds of poor MA to PERT compared to those with junior high school or lower education. Patients with monthly household income less than 5,000 (adj.OR = 4.16, 95% CI 1.36–12.75, P = 0.013) and 5000–10,000 (adj.OR = 2.94, 95% CI 1.01–8.58, P = 0.049) had higher odds of poor MA compared to those with monthly household income over 10,000.

**Table 3:** Multivariate logistic regression for factors influencing adherence to PERT among patients with CP.

Characteristics	adj.OR (95% CI)	*P* value
Age		
56–70	Reference	
36–55	2.89 (0.81, 10.35)	0.104
18–35	3.48 (0.87, 13.88)	0.077
Gender		
Male	Reference	
Female	1.04 (0.34, 3.13)	0.948
Education level		
Junior high school or below	Reference	
Senior high school	0.18 (0.06, 0.56)	**0.003**
University or above	0.32 (0.11, 0.94)	**0.039**
Employment		
Employed	Reference	
Unemployed/retired/in study	1.43 (0.50, 4.11)	0.504
Monthly household income (CNY)		
≥ 10,000	Reference	
5000–10,000	2.94 (1.01, 8.58)	**0.049**
< 5000	4.16 (1.36, 12.75)	**0.013**
Medical insurance type		
Employee medical insurance	Reference	
Urban residents' medical insurance	1.09 (0.35, 3.40)	0.882
Rural cooperative medical insurance	1.66 (0.63, 4.38)	0.307
History of smoking		
No	Reference	
Yes	0.528 (0.16, 1.82)	0.288
History of drinking		
No	Reference	
Yes	1.04 (0.31, 3.57)	0.945

adj OR, adjusted odds ratio; CNY, Chinese Yuan; PERT, pancreatic enzyme replacement therapy.

Significant values are in bold.

### Qualitative analysis

#### Characteristics of participants

27 of the 148 patients were invited to participate in the interview, and three of them declined. Table 4 showed the characteristics of the 24 interviewed participants. Supplement Table 1 showed the detailed information of each interviewed participate. Among them, 14 (58.33%) were male, age ranged 25–59 years, the mean score of C-MMAS-8 was 5.25 ± 1.99, and 16 (66.67%) had poor adherence to PERT.

**Table 4:** Characteristics of interviewed participants (n = 24).

Characteristics	n (%)
Age, mean (SD)	39.71 (9.48)
Gender, n (%)	
Male	14 (58.33)
Female	10 (41.67)
Education level, n (%)	
Junior high school or below	4 (16.67)
Senior high school	10 (41.67)
University or above	10 (41.67)
Marital status, n (%)	
Married	17 (70.83)
Unmarried/divorced/widowed	7 (29.17)
Employment, n (%)	
Employed	16 (66.67)
Unemployed/retired/ in study	8 (33.33)
Monthly household income (CNY), n (%)	
< 5000	8 (33.33)
5000–10,000	9 (37.50)
≥ 10,000	7 (29.17)
Residence, n (%)	
Urban	15 (62.50)
Rural	9 (37.50)
Medical insurance type, n (%)	
Rural cooperative medical insurance	6 (25.00)
Urban residents' medical insurance	9 (37.50)
Employee medical insurance	9 (37.50)
Course of disease, mean (SD)	5.67 (5.50)
Type of pain after taking medication	
RAP/RAP + RP	17 (70.83)
RP	4 (16.67)
Chronic pain	0 (0.00)
No pain	3 (12.50)
Diabetes, n (%)	11 (45.83)
Steatorrhea, n (%)	11 (45.83)
History of smoking, n (%)	12 (50.00)
History of drinking, n (%)	11 (45.83)
C-MMAS-8 score^a^	
Mean (SD)	5.25 (1.99)
< 6	12 (60.00)
6–< 8	6 (30.00)
8	2 (10.00)

CNY, Chinese Yuan; CP, chronic pancreatitis; RAP, repeat attacks of acute pancreatitis; RAP + RP, repeat acute attacks and pain; RP, repeat pain; SD, standard deviations.

*n = 20.

#### Key themes

Seven themes regarding barriers to MA emerged from interviews: (1) Lack of knowledge, (2) Self-adjustment of PERT, (3) Lifetime of medication, (4) Side effects of PERT, (5) Forgetfulness, (6) Financial burdens, and (7) Accessibility issues (Table 5).

**Table 5:** Themes and units of meaning.

Themes	Units of meaning
Lack of knowledge	*“I didn't know that I had to take this medicine all the time, and the doctor there didn't tell me. I stopped taking it, after eating up the medicine prescribed by the doctor every time.”* (N15)
	*“I have taken the medicine before, and the doctor said that the medicine needed to be taken for the rest of my life. I was worried that the long-term use of it would cause damage to my liver and kidneys, and then I stopped taking it later.”* (N24)
Self-adjustment of PERT	*“Usually, I did not take medicine if I felt fine, and if I felt my stomach rising or a little uncomfortable, I would take more pills on my own.”* (N7)
	*“If I ate porridge in the morning, I would not take the medicine, and if I went to a restaurant or ate greasy, or went out to socialize and drank, I would add a few pills myself.”* (N22)
Lifetime of medication	*“The doctor told me to take the drug for life, and the dosage could be a little less when I was young and increased when I was older. I took it for a while, but it didn't seem to have much effect, so I stopped taking it. I wanted to wait until I get older.”* (N5)
Side effects of PERT	*“Originally, I had symptoms of diarrhea and had to have bowel movements 3–4 times a day. However, once I took this medicine, I would become constipated, I may not have a bowel movement for several days, and my stomach felt bloated, so I just stopped taking it.”* (N10)
Forgetfulness	*“I am working as a salesman, and the nature of my work is different from that of others sitting in the office, with medicine on their desk. Sometimes I forgot to take my medicine when I go out. Sometimes I would forget to take medicine when I was busy or had irregular meals.”* (N14)
	*“This medicine should be taken at mealtime, and I easily forget to take it without being reminded. Sometimes I remembered it after meal.”* (N8, N23)
Financial burdens	*“I had to take four pills at a time, three times a day is 12 pills, and a bottle of medicine was finished within a few days. My wife and I were both part-time workers. We can't afford to eat so much, so we switched to domestic pancreatic enzyme tablets later.”* (N6)
Accessibility issues	*“This drug cannot be bought in our county at all, only the two hospitals in the provincial capital had the sale. Also, I cannot buy a lot at once. I can only buy 2 weeks of drugs at a time with my medical insurance reimbursement. In addition, it was inconvenient to travel due to the COVID-19 epidemic. It was too troublesome to buy medicine.”* (N20)

##### Theme 1: Lack of knowledge

Some patients reported that they were unaware of their disease condition, treatment options, and the nature of drugs they are taking. They believed they did not need to take medication or need to adhere to it for a long period of time.

*“I didn't know that I had to take this medicine all the time, and the doctor there didn't tell me. I stopped taking it, after eating up the medicine prescribed by the doctor every time.”* (N15)

*“I have taken the medicine before, and the doctor said that the medicine needed to be taken for the rest of my life. I was worried that the long-term use of it would cause damage to my liver and kidneys, and then I stopped taking it later.”* (N24)

##### Theme 2: Self-adjustment of PERT

Some patients will exert their subjective initiative in taking the medication and changed the dosage according to the severity of their symptoms and type of diet.*“Usually, I did not take medicine if I felt fine, and if I felt my stomach rising or a little uncomfortable, I would take more pills on my own.”* (N7)

*“If I ate porridge in the morning, I would not take the medicine, and if I went to a restaurant or ate greasy, or went out to socialize and drank, I would add a few pills myself.”* (N22)

##### Theme 3: Lifetime of medication

Since CP patients will not show obvious symptoms during the stable phase, the benefits of taking the medication cannot be clearly perceived in a short term and coupled with the fact that pancreatic enzyme need to be taken by patients for a long period of time, a long-term use of the medication may reduce patients’ MA.

*“The doctor told me to take the drug for life, and the dosage could be a little less when I was young and increased when I was older. I took it for a while, but it didn't seem to have much effect, so I stopped taking it. I wanted to wait until I get older.”* (N5)

##### Theme 4: Side effects of PERT

A few patients reported some side effects after taking the medication, which in severe cases affected their daily life, so they chose not to take the medication.

*“Originally, I had symptoms of diarrhea and had to have bowel movements 3–4 times a day. However, once I took this medicine, I would become constipated, I may not have a bowel movement for several days, and my stomach felt bloated, so I just stopped taking it.”* (N10)

##### Theme 5: Forgetfulness

Some patients said that they would forget to bring their medicines or forget to take drugs because they were busy at work or needed to go out. In addition, a small number of patients said that they relied on reminders from others to improve MA.

*“I am working as a salesman, and the nature of my work is different from that of others sitting in the office, with medicine on their desk. Sometimes I forgot to take my medicine when I go out. Sometimes I would forget to take medicine when I was busy or had irregular meals.”* (N14)

*“This medicine should be taken at mealtime, and I easily forget to take it without being reminded. Sometimes I remembered it after meal.”* (N8, N23)

##### Theme 6: Financial burdens

Some patients reported that taking medicine would cause a certain financial burden, so they chose not to take medicine or switch to other drugs.

*“I had to take four pills at a time, three times a day is 12 pills, and a bottle of medicine was finished within a few days. My wife and I were both part-time workers. We can't afford to eat so much, so we switched to domestic pancreatic enzyme tablets later.”* (N6)

##### Theme 7: Accessibility issues

Considering that CP is a rare disease, not all hospitals can provide the medications patients need. Some patients reported that they lacked access to medication.

*“This drug cannot be bought in our county at all, only the two hospitals in the provincial capital had the sale. Also, I cannot buy a lot at once. I can only buy 2 weeks of drugs at a time with my medical insurance reimbursement. In addition, it was inconvenient to travel due to the COVID-19 epidemic. It was too troublesome to buy medicine.”* (N20)

## Discussion

Although PERT has been recommended as the first choice for the treatment of PEI caused by CP**,** there are significant center related variances in the management of PERT in clinical practice, and many patients have not been treated in a standardized way^18^. In available studies, data on adherence to PERT among CP patients is limited. This may be very important as identifying the levels of and factors influencing MA may help to better implement PERT management and improve the prognosis of PEI. To our knowledge, this study was the first to explore the adherence to PERT among patients with CP in China. The mixed study design was also a strength of this paper, allowing for a more comprehensive perspective on the factors influencing MA. Overall, MA to PERT was currently poor in Chinese CP patients. During the qualitative interviews, we found that factors affecting MA included lack of knowledge, self-adjustment of PERT, lifetime of medication, side effects of PERT, forgetfulness, financial burdens, and accessibility issues.

In this study, the rate of non-adherence to PERT reached 48.0% and only 12.8% had good MA. This result was close to the adherence to PERT after 1 year follow-up in the study by Khandelwal et al.^23^. We believed that this result was true and consistent with the current status of long-term adherence to PERT in patients with CP. However, this figure was significantly lower than that in the studies by Barkin et al. and Crosby et al.^21,22^. In their studies, the rate of adherence to PERT respectively reached 50% and 79% in patients with pancreatic cancer and those undergoing total pancreatectomy with islet autotransplantation. One possible explanation for such high MA in their studies was that patients included in our study all chose to receive interventional treatment for pancreatic duct stones, and the serious condition of pancreatic cancer and painful and traumatic surgical treatment experiences of the patients in their studies may contribute to their higher medication consciousness and adherence to PERT.

Adherence to PERT in patients with CP in our study was also lower than in some common chronic diseases. In a meta-analysis by Khunti et al. involving over 300,000 patients with type 2 diabetes, the mean rate of poor MA was 37.8%^31^. Another meta-analysis by Durand et al. on MA in patients with refractory hypertension reported that the pooled rates of non-adherence was 31.2%^32^. Cancer, cardiovascular diseases, diabetes and chronic respiratory diseases have been listed by the World Health Organization (WHO) as the top four non-infectious diseases worldwide^33^. Compared with these common chronic diseases widely publicized and well-known by the individuals, given the rarity of CP, there is a clear lack of knowledge of CP among patients, as evidenced by the interviews in this study, where patients reported that they were not well informed about their conditions and the medication they were taking (N15, N24). Therefore, we believed that a lack of disease knowledge may be one of the potential reasons for the poor adherence to PERT among CP patients. In addition, inadequate and inconsistent medication guidance from health care providers may be another reason for poor MA. Existing studies have concluded that there is a lack of uniform consensus among gastroenterologists on the diagnosis and treatment of PEI and that only a small number of pancreatic specialists can surveil and treat PEI adequately compared to primary care providers^17,34^.

Therefore, we recommend, first, standardized guidelines and additional education are necessary for healthcare providers. National advanced pancreatic disease institutions should develop and promote PEI treatment guidelines applicable to CP patients in their country and conduct educational courses and guideline interpretations for non-pancreatologist on PEI and PERT to help them prescribe uniform and standardized plan of PERT. Second, it is positive for patients to be informed about the disease and the medication they are taking. For example, providing patients with information about prescribed medications has been shown to improve patients’ MA^35,36^. In addition, given that MA usually decreases over time, and given the long-term nature of PERT and the lack of short-term effects (N5), the follow-up monitoring and management of medication behavior is also integral to improving MA^37^. For example, electronic pill boxes^38^, electronic reminders^39,40^, smartphone applications^41,42^ and closer communication between doctors and patients^43^ are thought to help improve patients’ MA.

In this study, the results of multivariate analysis showed that lower levels of education and income were contributing factors for non-adherence to PERT. In addition, forgetfulness, side effects and the cost of medication were potential factors affecting patients’ MA. These findings fall within the WHO framework for MA and were consistent with previous research findings^44–46^.

Economic factors are considered one of the most important influences on patients’ MA. In the quantitative analysis, patients with poor MA were more likely to have lower monthly household income (< 5000 CNY). Excessive medical costs and financial burden may be the cause of poor MA. Therefore, for patients who may have financial difficulties, health care providers should offer them an alternative cheaper drug choice at the time of prescribing. In terms of medication guidance, doctors and nurses should personalize the medication strategy according to patients’ lifestyle and guide them to adjust the dosage reasonably according to their diet to reduce medication expenditure. For medical administrations and medical institutions, improving non-local medical insurance settlement services and reducing patients’ own expenses are effective means to improve patients’ MA. In addition, during the interviews we also found that even patients with stable jobs similarly reported difficulties with MA (N14). The type of work, work environment and working hours of patients may influence their willingness and behavior of taking medication, however, these factors have not been explored in depth in previous articles.

The level of education was another important factor influencing patients’ adherence to PERT. This is easy to understand, as patients who have received better education usually have better comprehension and acceptance of knowledge related to diseases and medications and have a richer knowledge reserve, resulting in their better MA. Meanwhile, existing views suggested that patients’ cognition of diseases (such as etiology, disease harms, outcome, and controllability) will directly influence their MA^47^. However, we also found an interesting phenomenon. Generally, younger patients usually have a higher level of education compared to older patients, so their adherence to PERT should have been better. However, in fact, we found that MA appeared to be worse in younger patients. The possible explanations were, first, younger patients had a relatively new diagnosis and a shorter course of disease, and that their relapses and disease experiences were less frequent. Second, young patients tended to have a better level of education. On the one hand, good education made it easier for them to understand and accept disease knowledge, but on the other hand, these patients were also relatively more rebellious, more likely to exert their subjective initiative in the process of taking drugs, and did not comply with drug prescriptions^48,49^. Considering the youthfulness and good acceptance of medication non-adherent patients, with the rapid development of new media technology and instant music video platforms, based on the existing e-reminders, smartphone applications and social platforms, video pushing of medication instructions on instant music video platforms such as TikTok and bilibili may be a new way to improve the MA of patients.

This study also had some limitations. First, this was a small sample study based on a single center. Although Changhai Hospital is the largest CP diagnosis and treatment center in China and even Asia, patients at our center are more representative only for East China. Small sample may also lead to some bias in the results of this study. In addition, patients who visited our center tended to be more severely ill and had better adherence than those in primary medical institutions. Therefore, the findings of our study may not be fully representative of the MA to PERT of other CP patients in other institutions. Second, we only used the C-MMAS-8 to measure patients’ MA and did not use other scales and data such as medication possession ratio and proportion of days covered for validation, which may lead to some bias of the MA results from the real situation. Third, although this study provided first-hand information on the adherence to PERT in Chinese CP patients, the limited number of factors influencing patients’ MA included in the analysis may have led to the results of this study was not novel. It is expected that the future studies can, based on our research, cooperate with more centers of different scales in China to carry out extensive and large-sample research, adopt multiple MA measurement methods, and incorporate more indicators such as patients’ disease knowledge, health literacy, self-efficacy, and indicators of patients’ exocrine pancreatic function et al. to further explore various subjective, objective, and disease-related factors of adherence to PERT in Chinese CP patients and validate the results of our study, providing a reference for the management of PERT.

## Conclusions

This study revealed the status of adherence to PERT among patients with CP in East China through a mixed study design. Overall, the MA of East Chinese CP patients was poor, and the low education and income level were the contributing factors of poor MA. The qualitative analysis results showed that, seven themes associated with non-adherence included lack of knowledge, self-adjustment of PERT, lifetime of medication, side effects of PERT, forgetfulness, financial burdens, and accessibility issues. Healthcare providers should personalize medication strategies to improve the MA of patients.

### Supplementary Information

Supplementary Tables.

## Data Availability

The datasets used and/or analyzed during the current study are available from the corresponding author on reasonable request.

## Abbreviations

CNY: Chinese Yuan
CP: Chronic pancreatitis
MA: Medication adherence
MMAS-8: Morisky Medication Adherence Scale
PEI: Pancreatic exocrine insufficiency
PERT: Pancreatic enzyme replacement therapy
