# Supplementary material for: Adherence to pancreatic enzyme replacement therapy among patients with chronic pancreatitis in East China: a mixed methods study
Source: Sci Rep. 2023 Oct 10;13:17147. doi: 10.1038/s41598-023-44519-3 (PMC10564898; doi:10.1038/s41598-023-44519-3)
Supplement: Supplementary file 1 — Supplementary Tables. [file 41598_2023_44519_MOESM1_ESM.docx]

**Supplement Table 1. Detailed information of each interviewed participate.**

| Participant | Gender | Age | Education level | Employment | Monthly household income (CNY) | Medical insurance type | Course of disease | Type of pain after taking medication | C-MMAS-8 score |
| --- | --- | --- | --- | --- | --- | --- | --- | --- | --- |
| N1 | Male | 58 | University or above | Retired | ≥10,000 | Employee medical insurance | 13 | RP | 7 |
| N2 | Male | 32 | Senior high school | Employed | <5,000 | Urban residents' medical insurance | 1 | RAP | 5.5 |
| N3 | Male | 50 | Senior high school | Unemployed | 5,000-10,000 | Rural cooperative medical insurance | 1 | RP | 7 |
| N4 | Female | 25 | Senior high school | Employed | <5,000 | Rural cooperative medical insurance | 9 | RAP | 3.75 |
| N5 | Female | 26 | University or above | Employed | 5,000-10,000 | Employee medical insurance | 5 | RAP | NA |
| N6 | Male | 50 | Junior high school | Unemployed | <5,000 | Rural cooperative medical insurance | 14 | RAP+RP | 3.75 |
| N7 | Male | 49 | University or above | Unemployed | <5,000 | Urban residents' medical insurance | 12 | RAP | 2.75 |
| N8 | Female | 46 | University or above | Employed | 5,000-10,000 | Employee medical insurance | 1 | No pain | 6.75 |
| N9 | Male | 32 | Junior high school | Unemployed | <5,000 | Rural cooperative medical insurance | 8 | RAP+RP | 4.25 |
| N10 | Female | 37 | University or above | Employed | <5,000 | Urban residents' medical insurance | 5 | RAP | NA |
| N11 | Male | 42 | Senior high school | Employed | 5,000-10,000 | Employee medical insurance | 3 | RAP+RP | 8 |
| N12 | Male | 40 | Senior high school | Employed | 5,000-10,000 | Employee medical insurance | 22 | RAP+RP | 2.75 |
| N13 | Female | 50 | Senior high school | Employed | 5,000-10,000 | Urban residents' medical insurance | 1 | RP | 6.75 |
| N14 | Male | 34 | University or above | Employed | ≥10,000 | Urban residents' medical insurance | 12 | RAP+RP | 4.5 |
| N15 | Male | 32 | Junior high school | Employed | <5,000 | Rural cooperative medical insurance | 2 | RAP | NA |
| N16 | Male | 40 | Senior high school | Employed | ≥10,000 | Employee medical insurance | 2 | No pain | 8 |
| N17 | Female | 36 | University or above | Employed | ≥10,000 | Employee medical insurance | 1 | RAP+RP | 4.5 |
| N18 | Male | 42 | Senior high school | Employed | ≥10,000 | Urban residents' medical insurance | 3 | RP | 3.5 |
| N19 | Female | 35 | University or above | Employed | ≥10,000 | Employee medical insurance | 3 | No pain | 7.75 |
| N20 | Male | 26 | Senior high school | Unemployed | <5,000 | Rural cooperative medical insurance | 2 | RAP+RP | 1.5 |
| N21 | Female | 32 | Senior high school | Employed | 5,000-10,000 | Employee medical insurance | 8 | RAP | 4.5 |
| N22 | Male | 41 | University or above | Employed | ≥10,000 | Urban residents' medical insurance | 3 | RAP+RP | 7.75 |
| N23 | Female | 39 | Junior high school | Unemployed | 5,000-10,000 | Urban residents' medical insurance | 4 | RAP+RP | 4.75 |
| N24 | Female | 59 | University or above | Retired | 5,000-10,000 | Urban residents' medical insurance | 1 | RAP | NA |

Abbreviations: CNY, Chinese Yuan; CP, chronic pancreatitis; NA, not available; RAP, repeat attacks of acute pancreatitis; RAP+RP, repeat acute attacks and pain; RP, repeat pain.

**Supplement Table 2. COREQ (Consolidated criteria for reporting qualitative research) checklist.**

| **No.** | **Item** | **Guide questions/Description** | **Page No.** |
| --- | --- | --- | --- |
| **Domain 1: Research team and reflexivity** | | | |
| Personal Characteristics | | | |
| 1 | Interviewer/facilitator | Which author/s conducted the interview or focus group? | Page 6 |
| 2 | Credentials | What were the researcher’s credentials? E.g. PhD, MD | Page 6 |
| 3 | Occupation | What was their occupation at the time of the study? | Title page |
| 4 | Gender | Was the researcher male or female? | N/A |
| 5 | Experience and training | What experience or training did the researcher have? | Page 6 |
| Relationship with participants | | | |
| 6 | Relationship established | Was a relationship established prior to study commencement? | No |
| 7 | Participant knowledge of the interviewer | What did the participants know about the researcher? e.g. personal goals, reasons for doing the  research | Page 6 |
| 8 | Interviewer characteristics | What characteristics were reported about the interviewer/facilitator? e.g. Bias, assumptions, reasons and interests in the research topic | Page 5 |
| **Domain 2: study design** | | | |
| Theoretical framework | | | |
| 9 | Methodological orientation and Theory | What methodological orientation was stated to underpin the study? e.g. grounded theory, discourse analysis, ethnography, phenomenology, content analysis | N/A |
| Participant selection | | | |
| 10 | Sampling | How were participants selected? e.g. purposive, convenience, consecutive, snowball | Page 6 |
| 11 | Method of approach | How were participants approached? e.g. face-to-face, telephone, mail, email | Page 6 |
| 12 | Sample size | How many participants were in the study? | Page 7-8 |
| 13 | Non-participation | How many people refused to participate or dropped out? Reasons? | Page 8 |
| Setting | | | |
| 14 | Setting of data collection | Where was the data collected? e.g. home, clinic, workplace | Page 6 |
| 15 | Presence of non-participants | Was anyone else present besides the participants and researchers? | Page 7 |
| 16 | Description of sample | What are the important characteristics of the sample? e.g. demographic data, date | Page 8,  Table 4 |
| Data collection | | | |
| 17 | Interview guide | Were questions, prompts, guides provided by the authors? Was it pilot tested? | Page 6 |
| 18 | Repeat interviews | Were repeat interviews carried out? If yes, how many? | No |
| 19 | Audio/visual recording | Did the research use audio or visual recording to collect the data? | Page 6 |
| 20 | Field notes | Were field notes made during and/or after the interview or focus group? | Page 6 |
| 21 | Duration | What was the duration of the interviews or focus group? | Page 6 |
| 22 | Data saturation | Was data saturation discussed? | Page 6-7 |
| 23 | Transcripts returned | Were transcripts returned to participants for comment and/or correction? | No |
| **Domain 3: analysis and findings** | | | |
| Data analysis | | | |
| 24 | Number of data coders | How many data coders coded the data? | Page 7 |
| 25 | Description of the coding tree | Did authors provide a description of the coding tree? | No |
| 26 | Derivation of themes | Were themes identified in advance or derived from the data? | 7 |
| 27 | Software | What software, if applicable, was used to manage the data? | No |
| 28 | Participant checking | Did participants provide feedback on the findings? | No |
| Reporting | | | |
| 29 | Quotations presented | Were participant quotations presented to illustrate the themes / findings? Was each quotation identified? e.g. participant number | Page 11-14 |
| 30 | Data and findings consistent | Was there consistency between the data presented and the findings? | Page 7-10 |
| 31 | Clarity of major themes | Were major themes clearly presented in the findings? | Page 8-10,  Table 5 |
| 32 | Clarity of minor themes | Is there a description of diverse cases or discussion of minor themes? | N/A |

Developed from: Tong A, Sainsbury P, Craig J. Consolidated criteria for reporting qualitative research (COREQ): A 32-item checklist for interviews and focus groups. *Int J Qual Heal Care*. 2007;19(6):349-357. doi:10.1093/intqhc/mzm042
